# Early childhood exposure to environmental phenols and parabens, phthalates, organophosphate pesticides, and trace elements in association with attention deficit hyperactivity disorder (ADHD) symptoms in the CHARGE study

**DOI:** 10.21203/rs.3.rs-2565914/v1

**Published:** 2023-02-10

**Authors:** Jiwon Oh, Kyoungmi Kim, Kurunthachalam Kannan, Patrick J. Parsons, Agnieszka Mlodnicka, Rebecca J. Schmidt, Julie B. Schweitzer, Irva Hertz-Picciotto, Deborah H. Bennett

**Affiliations:** University of California, Davis; University of California, Davis; New York University; Wadsworth Center; University of California, Davis; University of California, Davis; University of California, Davis; University of California, Davis; University of California, Davis

**Keywords:** ADHD, environmental phenols, parabens, phthalates, organophosphate pesticides, trace elements, mixtures

## Abstract

**Background:**

Agrowing body of literature investigated childhood exposure to environmental chemicals in association with attention deficit hyperactivity disorder (ADHD) symptoms, but limited studies considered urinary mixtures of multiple chemical classes. This study examined associations of concurrent exposure to non-persistent chemicals with ADHD symptoms in children diagnosed with autism spectrum disorder (ASD), developmental delay, and typical development.

**Methods:**

A total of 574 children aged 2–5 years from the Childhood Autism Risks from Genetics and Environment (CHARGE) case-control study was administered the Aberrant Behavior Checklist (ABC). This study focused on the Hyperactivity subscale and its two subdomains (hyperactivity/impulsivity, inattention). Sixty-two chemicals from four classes (phenols/parabens, phthalates, organophosphate pesticides, trace elements) were quantified in child urine samples, and 43 chemicals detected in >70% samples were used in statistical analyses. Weighted quantile sum regression for negative binomial outcomes with repeated holdout validation was performed to investigate covariate-adjusted associations between mixtures and ABC scores in 574 children. The mixture analyses were further restricted to 232 children with ASD.

**Results:**

Phthalate metabolite mixtures, weighted for mono-n-butylphthalate (MNBP), mono-2-heptyl phthalate, and mono-carboxy isononyl phthalate, were associated with the Hyperactivity subscale (mean incidence rate ratio [mIRR] = 1.11; 2.5th, 97.5th percentile: 1.00, 1.23), especially the hyperactivity/impulsivity subdomain (mIRR = 1.14; 2.5th, 97.5th percentile: 1.06, 1.26). These associations remained similar after restricting to children with ASD. The inattention subdomain was associated with a phenols/parabens mixture, weighted for several parabens and bisphenols (mIRR = 1.13; 2.5th, 97.5th percentile: 1.00, 1.28) and a total mixture, weighted for 3,4-dihydroxy benzoic acid, MNBR and mono-(2-ethyl-5-carboxypentyl) phthalate (mIRR = 1.11; 2.5th, 97.5th percentile: 1.01,1.25) only among children with ASD.

**Conclusions:**

Concurrent exposure to phthalate mixtures was associated with hyperactivity in early childhood. Though causal inference cannot be made based on our cross-sectional findings, this study warrants further research on mixtures of larger number of chemicals from multiple classes in association with ADHD-related behaviors in young children.

## 1. Background

Attention deficit hyperactivity disorder (ADHD) is a neurodevelopmental disorder, in which the individual manifests developmentally inappropriate levels of symptoms of inattention and/or hyperactivity/impulsivity (American Psychiatric Association 2013). Symptoms associated with the disorder occur on a continuum. ADHD is highly common, though the prevalence rates of ADHD vary depending on the methods used to assess presence of the disorder, with estimates ranging from 5.9% of youth meeting criteria (Willcutt 2012) to 9.4% (Danielson et al. 2018), with a sex ratio of 2.3 to 1 for males to female children (Willcutt 2012). Because ADHD symptoms are increasingly diagnosed in the autism population, with the publication of the Diagnostic and Statistical Manual-5 (DSM-5) edition permitting the co-morbid diagnosis of ADHD to be given in autistic individuals (American Psychiatric Association 2013), it is important to understand what factors might influence the presence of ADHD symptoms in autistic as well as non-autistic individuals. Estimates of ADHD symptoms in autism vary with older studies finding lower estimates, for example 2% (Hanson et al. 2013), and more recent studies, as high as 78% (Brookman-Frazee et al. 2018). The importance of studying autistic youth with significant ADHD symptoms is reinforced by findings from a recent study indicating 1.2% of children in the U.S. have both disorders (Casseus 2022). Our group found that the rate of ADHD symptoms in children diagnosed with autism as well as with neurodevelopmental disorders who do not have autism is significantly higher than expected in the general population (Lyall et al. 2017).

Despite the high heritability of ADHD, environmental factors, including chemical exposures, nutrient deficiencies, preterm birth, pregnancy complications, and extreme deprivation, are also associated with development of ADHD (Banerjee et al. 2007; Faraone et al. 2021). An accumulating body of epidemiological literature suggests that prenatal and early-life exposures to environmental chemicals are associated with ADHD diagnosis or symptoms (Moore et al. 2022; Schantz et al. 2020).

Environmental phenols and parabens are widely applied to household and personal care products, food and food contact materials, and pharmaceuticals (Buckley et al. 2020; Sakhi et al. 2018). Several environmental phenols and parabens can disrupt endocrine functions due to their structural similarity to hormones (Le Maire et al. 2010; Mitra et al. 2021), which may induce abnormal behavioral changes (Masuo and Ishido 2011). Previous epidemiological studies focusing on prenatal or childhood exposure to bisphenol A (BPA) and parabens in association with ADHD diagnosis or symptoms, have reported mixed results. Several studies observed adverse associations of ADHD diagnosis or related behaviors in middle childhood with BPA exposure during pregnancy (Casas et al. 2015; Jedynak et al. 2021) or childhood (Li et al. 2018b; Tewar et al. 2016; Yoo et al. 2020), while others reported null associations (Arbuckle et al. 2016; Guo et al. 2020; Hansen et al. 2021; Minatoya et al. 2018; Perez-Lobato et al. 2016; Roen et al. 2015; Skarha et al. 2020). For parabens, diagnosis of ADHD was associated with prenatal exposure to methyl paraben (MEPB) (Baker et al. 2020) or childhood exposure to ethyl paraben (ETPB) among males only (Tsai et al. 2020). A limited number of studies reported mixed associations between ADHD-related behaviors and prenatal or childhood exposure to other phenols, such as triclosan (TCS), benzophenone-3, and dichlorophenols (Etzel et al. 2018; Guo et al. 2020; Jackson-Browne et al. 2019; Jedynak et al. 2021; Philippat et al. 2017).

Phthalates are high-production-volume chemicals ubiquitously used in children’s products, personal care products, polyvinyl chloride flooring and plastics, food packaging, and medical devices (Buckley et al. 2020; Hauser and Calafat 2005; Heudorf et al. 2007). Phthalates can exert neurotoxicity by disrupting thyroid hormone homeostasis, leading to behavioral problems and impaired cognition (Ghassabian and Trasande 2018; Hlisníková et al. 2021; Miodovnik et al. 2014). A large body of epidemiological evidence reported that phthalate exposure during pregnancy (Chen et al. 2019; Day et al. 2021; Engel et al. 2010; Engel et al. 2018; England-Mason et al. 2020; Huang et al. 2019; Hyland et al. 2019; Jedynak et al. 2021; Kamai et al. 2021; Kobrosly et al. 2014; Li et al. 2020b; Lien et al. 2015; Minatoya et al. 2018; Watkins et al. 2021) or childhood (Arbuckle et al. 2016; Chopra et al. 2014; Hu et al. 2017; Jankowska et al. 2019b; Kim et al. 2009; Ku et al. 2020; Li et al. 2020b; Park et al. 2015; Tsai et al. 2020; Won et al. 2016) was adversely associated with ADHD diagnosis or related behaviors. A few studies observed null (Jankowska et al. 2019a) or favorable associations with phthalate exposure (Daniel et al. 2020; Gascon et al. 2015; Philippat et al. 2017).

Organophosphate (OP) pesticides are applied to agricultural and residential settings, and their residue on foods is the primary exposure source (Barr et al. 2004; Cequier et al. 2017; Holme et al. 2016). OP pesticides could adversely affect neurodevelopment primarily by inhibiting the enzyme acetylcholinesterase in the brain, which may lead to neurodevelopmental disorders as well as behavioral and cognitive deficits (Hertz-Picciotto et al. 2018; Richardson et al. 2019). However, limited studies reported prenatal or childhood exposure to OP pesticides, specifically dialkyl phosphate (DAP) metabolites, in association with ADHD diagnosis or symptoms, showing adverse (Bouchard et al. 2010; Marks et al. 2010; Sagiv et al. 2021; Yu et al. 2016) or null associations (Eskenazi et al. 2007; Furlong et al. 2017; Jedynak et al. 2021; Manley et al. 2022; Oulhote and Bouchard 2013; van den Dries et al. 2019).

Trace elements are naturally occurring elements present in environmental matrices (Tchounwou et al. 2012). Lead and mercury are known neurotoxicants (Davidson et al. 2004; Mason et al. 2014), but they are generally measured in whole blood for epidemiological studies (Kim et al. 2020a). Several other trace elements, such as arsenic (As), cadmium (Cd), or thallium (Tl), which are routinely measured in urine, also have neurotoxicity in laboratory settings (Dinocourt et al. 2015; Grandjean and Herz 2015; Osorio-Rico et al. 2017), but epidemiologic evidence in human studies are inconclusive. For instance, prenatal or childhood exposure to As, Cd, or Tl showed adverse associations with ADHD diagnosis or related behaviors in some studies (Bao et al. 2009; Gustin et al. 2018; Kim et al. 2020b; Renzetti et al. 2021 a; Roy et al. 2011; Sioen et al. 2013; Skogheim et al. 2021; Tong et al. 2020), but others did not observe convincing associations (Ciesielski et al. 2012; Forns et al. 2014; Jedynak et al. 2021; Kim et al. 2013; Yousef et al. 2011). Associations of other trace elements, specifically beryllium and uranium, with ADHD-related behaviors are rarely studied.

Young children are exposed to mixtures of non-persistent environmental chemicals, including environmental phenols and parabens, phthalates, OP pesticides, and trace elements (CDC 2022). However, limited studies focused on ADHD-related behaviors in association with mixtures of multiple classes of urinary chemicals to address real-world exposures (Guilbert et al. 2021; Maitre et al. 2021; van den Dries et al. 2021; Waits et al. 2022). This study aimed to examine if concurrent exposure to each chemical as well as mixture of these chemicals is associated with ADHD symptoms in early childhood in a cohort that includes children diagnosed with autism spectrum disorder (ASD), developmental delay (DD), and typical development (TD).

## 2. Methods

### 2.1. Study population

Our study population consisted of a subset of children from the Childhood Autism Risks from Genetics and Environment (CHARGE) case-control study (Hertz-Picciotto et al. 2006). The CHARGE study primarily recruited children who received services for ASD or DD through the California Department of Developmental Services. General population controls were randomly selected from state birth files and frequency-matched to the sex, age, and residential catchment area of ASD cases. Given the male-to-female ASD prevalence ratio, the goal was to recruit more males (80%) than females (20%). Children were eligible for inclusion in the CHARGE study if they were 2 to 5 years old at enrollment, born in California, living with at least one biologic parent who speaks English or Spanish, and residing in the study catchment areas. Details on study design, subject recruitment, and data collection protocols are available elsewhere (Hertz-Picciotto et al. 2006). The diagnosis of children obtained from recruitment process was confirmed using a set of standardized clinical assessments. Diagnostic tools and algorithms to classify children into ASD, DD, or TD group are described elsewhere (Hertz-Picciotto et al. 2006). Among those who were enrolled between 2006 and 2017, a total of 574 children who provided a sufficient volume (≥ 16 ml) of urine and were diagnosed with either ASD (*n* = 232), DD (*n* = 94), or TD (*n* = 248) were included in this study.

### 2.2. Assessment of ADHD symptoms

Children at 2 to 5 years old were assessed for ADHD symptoms using the Aberrant Behavior Checklist (ABC) at the UC Davis Medical Investigations of Neurodevelopmental Disorders (MIND) Institute. The ABC was selected to assess behavioral symptoms because a substantial portion of the participants have intellectual disability. It was developed for children with intellectual disability but has good validity in children with ASD and TD as well as in toddlers at 14 to 43 months of age (Kaat et al. 2014; Karabekiroglu and Aman 2009). The ABC consists of 58 items, each of which is scored from 0 (not at all a problem) to 3 (the problem is severe in degree) with higher scores indicating more problems (Aman et al. 1985). Among five subscales (irritability, lethargy, stereotypy, hyperactivity, and inappropriate speech) derived from the items, the current study focused on the Hyperactivity subscale (16 items with a score range of 0–48) to assess ADHD symptoms. The Hyperactivity subscale was further separated into two subdomains to explore the ADHD symptoms by subtypes: hyperactivity/impulsivity (10 items with a score range of 0–30) and inattention (3 items with a score range of 0–9) (Lyall et al. 2017), while excluding the items related to defiance and oppositionality as typically those items are on separate scales in commonly used behavioral instruments, such as Child Behavior Checklist (Achenbach and Rescorla 2000) or Conners’ Parent Rating Scale (Conners 1997). The list of items that belong to the Hyperactivity subscale and two subdomains is shown in Table S1.

### 2.3. Urinary chemical quantification

Child spot urine samples were collected at the study visit when the child was 2 to 5 years of age. The urine samples were immediately stored at −20°C, and aliquots were shipped on dry ice to the New York State Department of Health’s Wadsworth Center’s Child Health Exposure Analysis Resource (CHEAR) Targeted Analysis Laboratory. A total of 62 trace organic chemicals were analyzed in the urine samples within the CHEAR organic biomonitoring section at Wadsworth: 30 phenols/parabens, 20 phthalate metabolites, 6 DAP metabolites of OP pesticides, and 6 trace elements. The chemical names and abbreviations of the 62 analytes are presented in Table S2. For analysis of phenols/parabens, urine samples were enzymatically deconjugated and extracted using liquid-liquid extraction and analyzed by high-performance liquid chromatography-tandem mass spectrometry (HPLC-MS/MS) (Asimakopoulos et al. 2014; Li et al. 2018a; Rocha et al. 2018). For quantification of phthalate metabolites, urine samples were processed using enzymatic deconjugation followed by solid-phase extraction (SPE) prior to HPLC-MS/MS analysis (Li et al. 2019; Rocha et al. 2017). DAP metabolites were extracted from urine samples using SPE and analyzed by HPLC-MS/MS (Li et al. 2020a). Trace elements in urine were analyzed within the CHEAR section of the Laboratory of Inorganic and Nuclear Chemistry at Wadsworth using Inductively Coupled Plasma Mass Spectrometry (ICP-MS) (Minnich et al. 2008). Detailed descriptions of the analytical method for each chemical class, including sample preparation, instrumental analysis, and mass spectrometric parameters, are available elsewhere (Bennett et al. 2022).

Fifteen blinded duplicates were analyzed with study samples, along with multiple CHEAR reference materials, for quality assurance purposes. Median relative percentage differences of the valid duplicate samples, in which both were greater than the limit of detection (LOD), ranged from 5–46% for phenols/parabens, 5–38% for phthalate metabolites, 8–13% for OP pesticide metabolites, and 1 −27% for trace elements (Table S3). The LODs ranged from 0.02 to 1 ng/mL for phenols/parabens, 0.01 to 5 ng/mL for phthalate metabolites, 0.02 to 0.1 ng/mL for pesticide metabolites, and 0.0007 to 0.45 ng/mL for trace elements (Table S3). Instrument software-generated values were used for urinary chemical concentrations below the LOD to reduce bias from replacing non-detected concentrations with a single value (Richardson and Ciampi 2003; Schisterman et al. 2006).

### 2.4. Statistical analysis

Among 62 analytes quantified in child urine samples, 43 chemicals with detection frequencies over 70%, including 21 phenols/parabens, 12 phthalates, 5 pesticides, and 5 trace elements, were included in the statistical analyses. Several zero or negative values, occurred as a result of blank correction of instrument software-generated values, were replaced with a fixed value (i.e., 0.0001) to allow natural log (ln)-transformation (Buckley et al. 2022). The positive nonzero values were then specific gravity (SG)-corrected using the following equation: *C*_*sg*_ = C × [(*SG*_*median*_ – 1)*/*(*SG* – 1), where *C*_*sg*_ is the SG-corrected chemical concentration, *C* is the measured chemical concentration, *SG*_*median*_ is the median (1.022) of SG values in this study samples, and *SG* is the measured SG value (Boeniger et al. 1993; Kuiper et al. 2021). The SG-corrected concentrations were In-transformed and standardized prior to the regression analyses to meet normality assumptions.

ABC Hyperactivity subscale and two subdomain scores and SG-corrected concentrations of representative compounds were compared by demographic characteristics using the Wilcox rank-sum or the Kruskal-Wallis test. MEPB, mono-isobutyl phthalate (MIBP), diethylphosphate (DEP), and uranium (U) were selected as representative compounds per each chemical class based on the high detection frequency and correlation with outcomes. Spearman’s correlation coefficients between compounds within each chemical class in the same CHARGE population were reported previously, showing moderate correlations among phthalate metabolites, OP pesticide metabolite, benzophenones, and parabens (Bennett et al. 2022).

Potential confounders and risk factors for ADHD were identified *a priori* based on a directed acyclic graph (Figure S1) (Hernán et al. 2004), and those variables that had associations with all three outcomes (*p* < 0.05) were selected as covariates. The final set of covariates includes: child’s sex (female, male), birth year (2000–2004,2005–2008,2009–2013), and age at assessment (in years; centered to the mean), maternal metabolic conditions (healthy weight/overweight and no metabolic conditions, obese or hypertensive disorder/gestational diabetes), parity (1, ≥ 2), highest education in household (high school/GED or less, some college credit, bachelor’s degree or higher), and diagnostic groups (ASD, DD, TD). Among the variables that reflect socioeconomic status and thereby are potentially correlated with each other, parental education was selected, instead of child’s race/ethnicity, mother’s age at delivery, and homeownership, to avoid overfitting issue because it was most strongly associated with both exposures and outcomes.

Negative binomial regression models, adjusting for the covariates, were used to examine the associations of each chemical with the ABC Hyperactivity subscale and two subdomain scores to account for excessive zeros in the outcomes. Incidence rate ratios (IRRs) and 95% confidence intervals (CIs) were estimated, and *p*-values were corrected for multiple comparisons using the false discovery rate (FDR) method per outcome and chemical class.

Repeated holdout validation for weighted quantile sum (WQS) regression for negative binomial outcomes was implemented to investigate the associations of each chemical class mixture with ABC scores (Tanner et al. 2019). For randomly partitioned training (40%) sets, the empirical weights, indicating relative importance, of each chemical were estimated across 100 bootstrap samples, and the WQS index, representing a total body burden, was computed per each chemical class using the estimated weights (Carrico et al. 2015; Lazarevic et al. 2019). The WQS index was used in negative binomial regression models with adjustment of the covariates to examine its association with the outcomes. We focused on the positive direction to make an inference regarding increased ABC scores in association with the mixture index. To obtain stable WQS estimates, the repeated holdout validation approach was used by randomly partitioning the dataset 100 times and performing the WQS regression on each set, generating 100 effect estimates and chemical weights and taking the mean as the final estimate (Tanner et al. 2019). Chemical weight distributions were presented when IRRs between 2.5th and 97.5th percentile (PCT) indicate significant associations (i.e., either IRR > 1 or IRR < 1). Chemicals that had 90% and 50% of repetitions above a threshold of each chemical class (1/number of chemicals in each mixture) were defined as probable and possible contributors, respectively (Bennett et al. 2022). Associations between total mixtures, encompassing all 43 analytes, and ABC scores were investigated using random subset WQS with repeated holdout validation, which iteratively selects random subsets of 7 chemicals (√43 ~ 7) and estimates weight parameters by combining results across multiple ensemble steps (Curtin et al. 2021; Tanner et al. 2019).

As children with ASD, followed by those with DD, showed more ADHD symptoms when compared to those with TD (Karabekiroglu and Aman 2009; Lyall et al. 2017), the mixture analyses were then restricted to children with ASD (*n* = 232) and with ASD or DD (*n=* 326). Furthermore, as previous studies reported sex-specific associations of phenols, phthalates, OP pesticides, and trace elements with child neurodevelopment (Bauer et al. 2020; Jankowska et al. 2021; Minatoya and Kishi 2021; Sapbamrer and Hongsibsong 2019; Schantz et al. 2020), child’s sex was evaluated as an effect modifier in the mixture models. Sex-stratified interaction WQS regression models that included the interaction term between sex and WQS index in addition to their main effects and covariates were constructed (Busgang et al. 2022; Gennings et al. 2022). From 100 repeated holdout datasets, distributions of sex-specific effect estimates and chemical weights were obtained. All analyses were performed with an open-source R software, version 4.1.0 (R Foundation for Statistical Computing, Vienna, Austria), including “gWQS” package (Renzetti et al. 2021 b). A statistical significance level was set at 0.05 for unadjusted *p*-values and 0.10 for FDR-corrected *p*-values.

## 3. Results

### 3.1. ABC scores by demographic characteristics

The majority of the study children were males (80.1 %) and born full-term (87.6%), and approximately 49% of them were non-Hispanic white (Table 1). More than half of the children were born to mothers who were not obese in pre-pregnancy and did not have any metabolic conditions (63.8%) and were multiparous (56.1 %) as well as to families whose highest education were bachelor’s degree or higher (56.6%) and owned a home (60.4%).

ABC Hyperactivity subscale and two subdomain scores differed by demographic characteristics (Table 1). Full-term children had lower ABC scores than children born pre-term, and non-Hispanic white or Hispanic children had lower scores compared to non-Hispanic, non-White (i.e., Asian, Black, and multiracial) children. Children whose mothers were 30 to 34 years old at delivery had lower scores than those whose mothers were younger than 30 years or at or older than 35 years. Children born to mothers who were obese in pre-pregnancy or had hypertensive disorder or gestational diabetes had higher scores compared to those born to mothers who were not obese or did not have metabolic conditions. The first-born children had higher scores than the second- or later-born children. Children born to parents whose maximum education level was high school or less had higher scores than those born to parents with higher education. Children from families that owned a home had lower scores than those from families that did not. In terms of diagnostic groups, children with ASD had the highest, those with DD had the second highest, and those with TD had the lowest scores.

### 3.2. Distributions of child urinary chemical concentrations and representative compounds by demographic characteristics

Detection frequency and distributions of SG-uncorrected concentrations of each chemical in child urine samples are presented in Table S3. Sixteen out of 30 phenols/parabens, 11 out of 20 phthalate metabolites, 5 out of 6 pesticide metabolites, and 4 out of 6 trace elements were detected in greater than 90% of the samples.

There were differences in urinary concentrations of representative compounds (i.e., MEPB, MIBR DER and U) by several characteristics (Table S4). For instance, children born in later years had higher DEP concentrations, and non-Hispanic, non-White children had higher MEPB concentrations. The first-born children had higher MEPB concentrations but tended to have lower MIBR DER and U concentrations. Children born to families with lower education levels had higher MIBP and U concentrations. The most prominent differences in urinary chemical concentrations were found across diagnostic groups: children with ASD had higher MIBR DER and U concentrations, and those with DD had higher MEPB concentrations.

### 3.3. Associations between individual chemical concentrations and ABC Hyperactivity subscale and two subdomain scores

**Figures 1:**
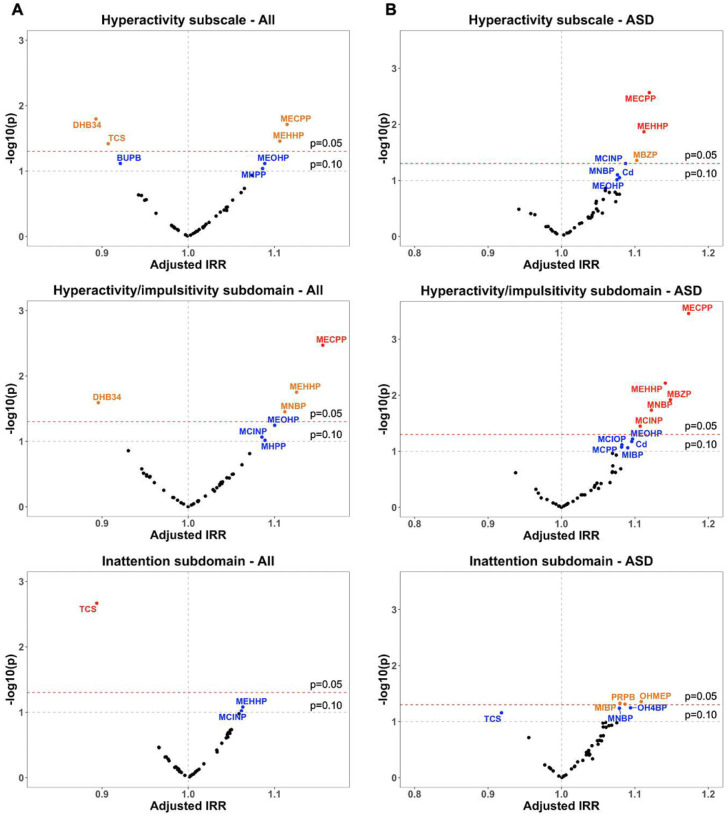
Volcano plots of covariate-adjusted IRRs and unadjusted *p*-values of SG-corrected, In-transformed, and standardized urinary chemical concentrations in association with ABC Hyperactivity subscale and two subdomain scores (A) among all children (*n*=574) and (B) children with ASD (*n*=232). Red dots represent chemicals with an unadjusted *p*<0.05 and an FDR-corrected *p*<0.10, orange dots represent chemicals with an unadjusted *p*<0.05, and blue dots represent chemicals with a 0.05<unadjusted *p* <0.10. Negative binomial regression models were adjusted for child’s sex, birth year, and age at assessment, parity, parental education, and maternal metabolic conditions. Models for all children were additionally adjusted for child’s diagnosis.

Volcano plots in Fig. 1 present significance and the magnitude of associations between individual urinary chemical concentrations and ABC scores. Among all children, including children with ASD, DD, and TD, increasing Hyperactivity subscale scores were significantly associated with mono-(2-ethyl-5-carboxypentyl) phthalate (MECPP) (IRR = 1.11, 95% Cl: 1.02,1.22) and mono (2-ethyl-5-hydroxyhexyl) phthalate (MEHHP) (IRR = 1.11, 95% Cl: 1.01,1.22) (Fig. 1A and Table S5). Decreasing Hyperactivity subscale scores were significantly associated with 3,4-dihydroxy benzoic acid (DHB34) (IRR = 0.89,95% Cl: 0.81, 0.98) and TCS (IRR = 0.91, 95% Cl: 0.82,1.00). Similar associations were observed for hyperactivity/impulsivity subdomain scores, except that the significant association with TCS was not found, while the adverse association with mono-n-butylphthalate (MNBP) was additionally observed (IRR = 1.11,95% Cl: 1.01,1.22). For the inattention subdomain, only TCS was significantly associated with decreasing scores (IRR = 0.89, 95% Cl: 0.83, 0.96). After correcting for FDR, associations of MECPP with the hyperactivity/impulsivity subdomain and TCS with the inattention subdomain remained significant (Table S5).

When restricting the analyses to children with ASD, adverse associations between other phthalate metabolites and ABC scores were additionally observed, including mono-benzyl phthalate (MBZP) with hyperactivity-related subscale (IRR = 1.10,95% Cl: 1.01,1.20) and the hyperactivity/impulsivity subdomain (IRR = 1.15, 95% Cl: 1.03,1.28), mono-carboxy isononyl phthalate (MCINP) with the hyperactivity/impulsivity subdomain (IRR = 1.11, 95% Cl: 1.01,1.21), and MIBP with the inattention subdomain (IRR = 1.08,95% Cl: 1.00,1.17) (Fig. 1B and Table S6). Increasing inattention subdomain scores were also associated with methyl-protocatechuic acid (OHMEP) (IRR = 1.11, 95% Cl: 1.00, 1.23) and propyl paraben (PRPB) (IRR = 1.09, 95% Cl: 1.00, 1.18) among children with ASD. Almost all the associations for the phthalate metabolites remained significant after FDR correction, while those for the phenols and parabens became non-significant at FDR < 0.1 (Table S6).

### 3.4. Associations between chemical class and total mixtures and ABC Hyperactivity subscale and two subdomain scores

**Figures 2:**
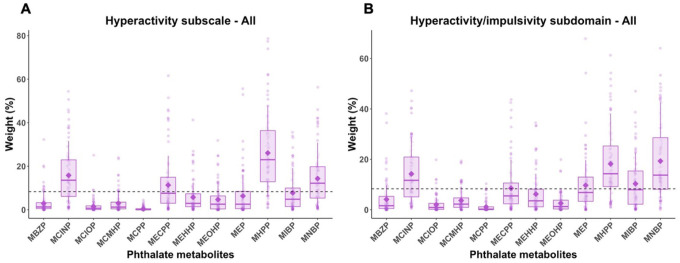
Estimated weight distributions of urinary phthalate metabolites from 100 repetitions of weighted quantile sum (WQS) regression for (A) Hyperactivity subscale and (B) hyperactivity/impulsivity subdomain, adjusting for child’s sex, birth year, and age at assessment, parity, parental education, maternal metabolic conditions, and diagnostic outcomes. Boxes indicate 25th, 50th, and 75th percentiles, and diamonds indicate mean, and whiskers indicate 10th and 90th percentiles of weights. Dashed line indicates a threshold (1/# of chemicals in the mixture).

Mixture analyses showed that the phthalate index was associated with increasing scores of the ABC Hyperactivity subscale (mean IRR = 1.11, 2.5th and 97.5th PCT: 1.00, 1.23) and the hyperactivity/impulsivity subdomain (mean IRR = 1.14, 2.5th and 97.5th PCT: 1.06, 1.26) (Table 2). MNBR mono-2-heptyl phthalate (MHPP), and MCINP were possibly contributed to the mixture effect on these subscale or subdomain scores (Fig. 2).

**Figures 3:**
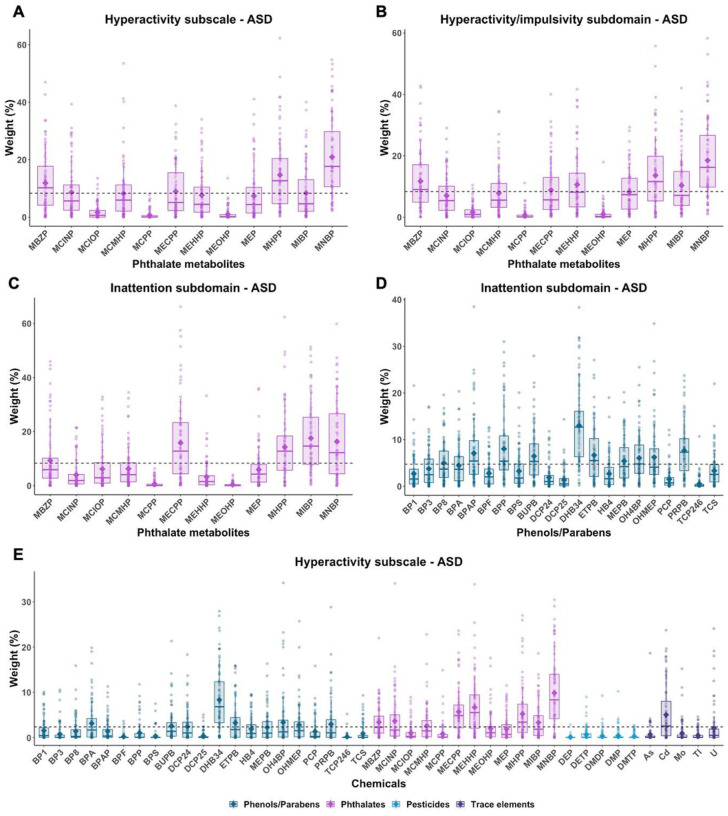
Estimated weight distributions of urinary chemicals from 100 repetitions of weighted quantile sum (WQS) regression, restricted to children with ASD. Phthalate metabolites in association with Hyperactivity subscale, hyperactivity/impulsivity subdomain, and inattention subdomain are presented in (A), (B), and (C), respectively. Phenols/parabens in association with inattention subdomain are presented in (D). Total chemicals in association with Hyperactivity subscale are presented in (E). Boxes indicate 25th, 50th, and 75th percentiles, and diamonds indicate mean, and whiskers indicate 10th and 90th percentiles of weights. Dashed line indicates a threshold (1/# of chemicals in the mixture).

When restricting the analyses to the children with ASD, the phthalate index was also associated with increasing scores of the inattention subdomain (mean IRR = 1.08, 2.5th and 97.5th PCT: 1.01, 1.17) as well as the Hyperactivity subscale and the hyperactivity/impulsivity subdomain (Table 2). While MNBR MHPR and MBZP were common possible contributors for hyperactivity-related subscale and subdomain, MNBR MHPR MIBR and MECPP were possible contributors for the inattention subdomain among children with ASD (Fig. 3). Furthermore, the phenols/parabens index, weighted with DHB34, ETPB, propyl paraben (PRPB), butyl paraben (BUPB), 4,4’-(1,4-phenylenediisopropylidene)bisphenol (BPP), and 4,4’-(1-phenylethylidene)bisphenol (BPAP), was associated with increasing inattention subdomain scores (mean IRR = 1.13, 2.5th and 97.5th PCT: 1.00, 1.28). The total mixture index was associated with increasing scores of all subscale and subdomains (mean IRR = 1.15 for Hyperactivity subscale, 1.21 for hyperactivity/impulsivity subdomain, 1.11 for inattention subdomain) (Table 2), and common contributors for the three scores were DHB34, MNBR and MECPP (Fig. 3 and Figure S2). When examined by subdomain, several phthalate metabolites, including MHPR MEHHR and MBZR and Cd were specifically weighted for the hyperactivity/impulsivity subdomain, while parabens, such as MEPB, PRPB, and OHMEP were specifically weighted for the inattention subdomain. The results restricted to the children with ASD or DD were similar to those from all children, but common possible contributors were MNBP and monoethyl phthalate (MEP) (Table S7 and Figure S3).

Sex-stratified interaction WQS regression models revealed that there was no significant effect modification by child’s sex for associations between any mixture and ABC scores (Table S8). However, the sex-specific associations of the phthalate index with hyperactivity-related subscale and subdomain were significant only among males. MNBR MHPR and MCINP were possibly contributed to these associations, as for all children (Figure S4).

## 4. Discussion

In the present study, concurrent measurement of environmental phenols and parabens, phthalates, OP pesticides, and trace elements in child urine samples were examined in association with ADHD symptoms, specifically the Hyperactivity subscale and the hyperactivity/impulsivity and inattention subdomains, among 2- to 5-year-old children diagnosed with either ASD, DD, or TD. For each compound, childhood MECPR one of the di-2-ethylhexyl phthalate (DEHP) metabolites, was cross-section ally associated with increased hyperactivity and impulsivity, while TCS with decreased inattention (Table 3). Concurrent exposure to phthalate mixtures was associated with hyperactivity, especially hyperactivity and impulsivity. These associations were possibly contributed by MNBR MHPR and MCINP and more strongly observed among males. However, as there was no effect modification by sex for these associations and this study population includes four times more males than females, which allowed greater statistical power, sex-specific associations should be interpreted with caution. Similar associations remained after restricting to children with ASD, but MNBR MHPR and MBZP were possible contributors. Only among children with ASD, the inattention subdomain was associated with a mixture of phthalate metabolites, possibly contributed by MNBR MHPR MIBR and MECPR and a mixture of phenols and parabens, possibly contributed by DHB34, ETPB, PRPB, BUPB, BPR and BPAP. Further, total mixtures of 43 urinary chemicals were associated with hyperactivity and two subdomains, and common possible contributors were DHB34, MNBR and MECPP.

Our findings on associations between childhood phthalate exposure, as an individual compound or a mixture, and greater ADHD symptoms in young children are in generally line with previous studies. One of the studies on childhood phthalate exposure in association with ADHD-related behaviors reported that MNBP and MEP as well as phthalate metabolite mixtures, possibly contributed by MCINR MER and MBZR were associated with more externalizing problems, indicating more hyperactivity, aggression, and conduct problems in children aged 2–8 (Li et al. 2020b). Another study observed cross-sectional associations of greater ADHD traits with MBZP at 2 years (Ku et al. 2020). Most of other studies examining ADHD diagnosis or related behaviors in middle-childhood or adolescence reported adverse associations with DEHP metabolites (Chopra et al. 2014; Hu et al. 2017; Kim et al. 2009; Park et al. 2015; Watkins et al. 2021) and di-n-butyl phthalate metabolites (Arbuckle et al. 2016; Jankowska et al. 2019b; Kim et al. 2009; Park et al. 2015; Tsai et al. 2020; Won et al. 2016), while few studies did not find convincing associations (Daniel et al. 2020; Huang et al. 2019; Jankowska et al. 2019a). Meanwhile, previous studies investigating ADHD diagnosis or symptoms in early childhood mainly focused on their associations with prenatal phthalate exposure, most of which observed adverse associations (Arbuckle et al. 2016; Day et al. 2021; Engel et al. 2010; Engel et al. 2018; England-Mason et al. 2020; Ku et al. 2020; Li et al. 2020b; Minatoya et al. 2018). However, young children not only have different exposure patterns to phthalates from their mothers, as indicated by weak correlations of phthalate metabolite concentrations in young children with those in their mothers’ prenatal or postnatal urine samples (Myridakis et al. 2015; Song et al. 2013), but also higher body burden (Choi et al. 2017; Wang et al. 2019). Therefore, this study warrants further investigations on early childhood exposure to phthalates and ADHD-related behaviors.

Underlying mechanisms of phthalates’ effects on ADHD remained unclear. ADHD is associated with alterations in the dopamine system and associated brain regions, such as the striatum and putatively, the midbrain (Elliott et al. 2022; Kowalczyk et al. 2022; Rosch et al. 2018; Shvarzman et al. 2022; Swanson et al. 2007). Toxicological studies reported that rats or mice neonatally exposed to DEHP or dicyclohexyl phthalate impaired tyrosine hydroxylase immunoreactivity within midbrain dopaminergic nuclei (Ishido et al. 2004; Tanida et al. 2009). Neonatal exposure of rats to DEHP or dibutyl phthalate expressed hyperactivity, concomitantly with alterations in gene expression in the midbrain and striatum (Ishido et al. 2005; Masuo et al. 2004a; Masuo et al. 2004b). Furthermore, cortical thickness is modestly thinner in children with ADHD and delayed in maturation in comparison to control participants (Bernanke et al. 2022; Shaw et al. 2012). Among children with ADHD, DEHP metabolite concentrations were negatively correlated with cortical thickness in the right middle and superior temporal gyri, suggesting a possible role of DEHP in impaired brain structures (Park et al. 2015). Phthalates are also reported to interfere with thyroid functions, which are essential for normal brain development, in a sexually dimorphic manner (Ghassabian and Trasande 2018; Miodovnik et al. 2014), and early thyroid hormone disruption may contribute to the development of ADHD (Drover et al. 2019). Still, regarding phthalate exposure and ADHD-related behaviors, there is inconsistent evidence on effect modification by sex (Chopra et al. 2014; Hu et al. 2017; Jankowska et al. 2021; Li et al. 2020b; Tsai et al. 2020; Won et al. 2016) or mediation by thyroid hormone (Engel et al. 2018); therefore, these should be explored in future studies.

In the present study, children with ASD showed associations of phenols/parabens mixtures, possibly contributed by ETPB, PRPB, BUPB, DHB34, BPR and BPAR with more ADHD symptoms, especially inattention, which were not observed in all children. One possible reason for the heterogeneity is more pronounced ADHD symptoms among children with ASD, showing higher and more variable ABC scores compared to those with DD or TD. In addition, parabens are used in preservatives in personal care products and foodstuffs (Buckley et al. 2020) and two bisphenols (BPAR BPP) are specifically used in food-related products (Wang et al. 2020). Because children with ASD are likely to have different dietary habits, behaviors, and usage of personal care products (Bandini et al. 2010; Hertz-Picciotto et al. 2011) resulting in different exposure patterns to parabens and bisphenols, potential reverse causality cannot be ruled out. Further studies on phenols/parabens exposures in relation to diets and behaviors in children with ASD can help address these questions.

There are a limited number of studies examining associations of prenatal or childhood exposure to mixtures of multiple classes of urinary chemicals with ADHD diagnosis or related behaviors. Guilbert et al. who quantified phthalate metabolites, phenols, and parabens in prenatal maternal urine samples, observed that a chemical mixture, primarily weighted for benophenone-3, TCS, MEPB, ETPB, and several phthalate metabolites (diisononyl phthalate metabolites, di(isononyl)cyclohexane-1,2-dicarboxylate metabolites, MBZR MEP), was associated with more externalizing behaviors (Guilbert et al. 2021). Van den Dries et al. reported null associations of prenatal exposure to mixtures of phthalates, BPA, and OP pesticides with attention problems in children aged 6 years (van den Dries et al. 2021). Maitre et al. that measured pre- and postnatal environmental exposures from outdoor, indoor, chemical, lifestyle and social domains observed associations of prenatal DMP exposure with more externalizing symptoms, while those of childhood DMP exposure with less ADHD symptoms (Maitre et al. 2021). Waits et al. examined concurrent exposure to phthalates, OP pesticides, and nonylphenol in relation to ADHD diagnosis in children aged 4–15 years, observing associations of a chemical mixture, primarily contributed by two OP pesticide metabolites (DMR DEP) and two phthalate metabolites (MER MBZP), with increased odds of ADHD (Waits et al. 2022). Many of these chemicals, to which the general population is simultaneously exposed, have endocrine disrupting potentials and share common mechanisms, including disruption of thyroid and neurotransmitter functions (Darbre 2022; Ghassabian and Trasande 2018; Masuo and Ishido 2011; Schug et al. 2015), and concentrations of these chemicals frequently measured in the urine are correlated within and across class (Bennett et al. 2022; Guilbert et al. 2021; Kalloo et al. 2018; Lee et al. 2017; van den Dries et al. 2021; Waits et al. 2022). Therefore, mixture analyses using multiple chemical classes helps with understanding of mixture effects of environmental chemicals on child neurobehaviors.

This study was strengthened by quantification of 62 chemicals from four chemical groups in urine samples of young children. WQS was employed to examine associations of chemical mixtures with ADHD-related behaviors, allowing for modeling multiple chemical exposures, which were correlated with each other, and minimizing multiple comparisons problem. However, several limitations should be noted. First, due to the cross-sectional design, our results do not represent causal effects of childhood chemical exposures on ADHD symptoms. Second, this study also relied on concentrations of non-persistent chemicals measured in a spot urine sample, which reflect recent exposure. In young children, several phenols, phthalate metabolites, OP pesticides, and trace elements showed moderate reproducibility over short-term periods but reduced reproducibility over longer time frames (Bradman et al. 2013; Casas et al. 2018; Stacy et al. 2016; Stacy et al. 2017; Teitelbaum et al. 2008; Watkins et al. 2014; Yen et al. 2022). Third, as this study used child urine samples as an exposure matrix, instead of whole blood samples, several other trace elements, especially known neurotoxicants, were not able to be included as analytes. Fourth, though an array of sociodemographic variables were considered as covariates, there is potential residual confounding by unmeasured factors related to diet, lifestyle, or parental ADHD symptoms. Fifth, our results cannot be generalized to general population because approximately 57% of our study population included children with ASD or DD, who showed more ADHD symptoms than those with TD.

## 5. Conclusions

In the CHARGE population, comprised of 2- to 5-year-old children diagnosed with ASD, DD, and TD, concurrent exposure to phthalate mixtures, highly weighted for MNBP and MHPR was associated with more hyperactivity, possibly driven by children with ASD. Only among children with ASD, inattention problems were associated with mixtures of phenols and parabens, possibly contributed by several parabens and their metabolite (ETPB, PRPB, BUPB, DHB34) and two bisphenols (BPR BPAP), and total mixtures, primarily weighted for paraben metabolite (DHB34) and two phthalates (MNBR MECPP). Because children with ASD not only have more pronounced ADHD behaviors but also show different exposure patterns to non-persistent chemicals due to different diet and behaviors, further attention to exposure of these children to possible neurotoxicants are warranted. Future investigation on exposure to mixtures of larger number of chemicals that share similar exposure sources could better address real-world exposures, in association with ADHD symptoms.

## Figures and Tables

**Table 1:** Aberrant Behavior Checklist (ABC) Hyperactivity subscale and two subdomain scores by characteristics of 574 CHARGE children.

Characteristics^a^	All children (*n* = 574)		Aberrant Behavior Checklist (ABC)					
			Hyperactivity subscale^b^ (*n* = 515)		Hyperactivity/impulsivity subdomain^b^ (*n* = 520)		Inattention subdomain^b^ (*n* = 547)	
	Freq (%)	Median (IQR)	*p*-value^c^	Median (IQR)	*p*-value^c^	Median (IQR)	*p*-value^c^
Sex			0.48		0.41		0.72
Male	460 (80.1)	7.0 (1.0, 18.8)		4.0 (1.0, 11.0)		2.0 (0.0, 3.8)	
Female	114 (19.9)	6.0 (1.0, 19.0)		3.0 (0.0, 10.0)		2.0 (0.0, 4.0)	
Child’s birth year			0.008		0.004		0.008
2000–2004	160 (27.9)	5.0 (1.0, 14.5)		3.0 (0.0, 10.0)		1.0 (0.0, 3.0)	
2005–2008	227 (39.5)	6.0 (1.0, 15.0)		3.0 (0.0, 10.0)		1.0 (0.0, 3.0)	
2009–2013	187 (32.6)	10.0 (3.0, 20.5)		6.0 (1.5, 13.5)		2.0 (0.0, 4.0)	
Preterm birth (< 37 weeks)			0.06		0.08		0.07
No	503 (87.6)	7.0 (1.0, 17.5)		4.0 (0.0, 10.0)		1.5 (0.0, 4.0)	
Yes	60 (10.5)	10.0 (3.3, 24.0)		6.0 (1.0, 14.3)		2.0 (1.0, 4.0)	
Child’s race/ethnicity			0.01		0.01		0.04
White (non-Hispanic)	279 (48.6)	5.0 (1.0, 16.0)		3.0 (0.0, 10.0)		1.0 (0.0, 3.0)	
Non-White (non-Hispanic)	120 (20.9)	10.0 (2.0, 24.5)		6.0 (1.0, 16.0)		2.0 (0.0, 4.0)	
Hispanic (any race)	170 (29.6)	7.5 (1.0, 15.0)		4.0 (1.0, 10.0)		2.0 (0.0, 3.0)	
Mother’s age at delivery			0.004	0.009			0.008
< 30 years	255 (44.4)	8.0 (1.0, 20.5)		4.0 (1.0, 13.0)		1.0 (0.0, 4.0)	
30–34 years	179 (31.2)	4.0 (0.5, 12.5)		2.0 (0.0, 8.0)		1.0 (0.0, 3.0)	
≥ 35 years	140 (24.4)	10.0 (3.0, 17.0)		5.5 (1.3, 10.0)		2.0 (0.0, 4.0)	
Maternal metabolic conditions			0.03	0.03			0.03
Healthy weight/overweight and no metabolic conditions	366 (63.8)	6.0 (1.0, 16.0)		3.0 (0.0, 10.0)		1.0 (0.0, 3.0)	
Obese or hypertensive disorder/gestational diabetes	192 (33.4)	8.0 (2.0, 21.8)		6.0 (1.0, 14.0)		2.0 (0.0, 4.0)	
Parity			< 0.001	< 0.001			< 0.001
1	233 (40.6)	9.0 (3.0, 22.0)		6.0 (1.0, 14.0)		2.0 (0.0, 4.0)	
≥ 2	322 (56.1)	5.0 (1.0, 14.0)		2.0 (0.0, 9.3)		1.0 (0.0, 3.0)	
Highest education in household			< 0.001	< 0.001			< 0.001
High school/GED or less	60 (10.5)	20.5 (8.5, 28.0)		12.0 (5.0, 19.0)		3.0 (2.0, 5.0)	
Some college credit	189 (32.9)	7.0 (1.0, 16.8)		4.0 (1.0, 10.0)		2.0 (0.0, 3.0)	
Bachelor’s degree or higher	325 (56.6)	5.0 (1.0, 15.0)		3.0 (0.0, 10.0)		1.0 (0.0, 3.0)	
Homeowner			0.008	0.007			0.002
No	172 (30.0)	9.0 (2.0, 22.3)		5.0 (1.0, 15.0)		2.0 (0.0, 4.0)	
Yes	381 (66.4)	6.0 (1.0, 15.8)		3.0 (0.0, 10.0)		1.0 (0.0, 3.0)	
Diagnostic groups			< 0.001	< 0.001			< 0.001
ASD	232 (40.4)	19.0 (10.0, 27.0)		11.0 (5.0, 18.0)		4.0 (2.0, 5.0)	
DD	94 (16.4)	8.5 (3.5, 19.0)		5.0 (2.0, 11.5)		2.0 (1.0, 4.0)	
TD	248 (43.2)	1.0 (0.0, 5.0)		1.0 (0.0, 3.0)		0.0 (0.0, 1.0)	
	Mean (SD)	r_sp_^d^	*p*-value^e^	r_sp_^d^	*p*-value^e^	r_sp_^d^	*p*-value^e^
Child’s age at assessment	3.9 (0.7)	0.12	0.005	0.13	0.004	0.11	0.01

a Missing (*n)*: preterm birth (11), child’s race/ethnicity (5), maternal metabolic condition (16), parity (19), homeowner (21)

b Mean ± SD for each subscale or subdomain score in the whole study population: 10.8 ± 11.4 for Hyperactivity subscale, 6.9 ± 7.6 for hyperactivity/impulsivity subdomain, 2.2 ± 2.3 for inattention subdomain

c P-values from the Wilcox rank-sum test or the Kruskal-Wallis test.

d Spearman’s rank correlation coefficients between child’s age and ABC scores or DEP concentrations.

e P-values from the significance test of Spearman’s rank correlation coefficient.

**Table 2:** Covariate-adjusted associations between mixtures and ABC Hyperactivity subscale and two subdomain scores among all children and children with ASD.

Outcome	Mixture	All (*n* = 574)			ASD (*n* = 232)		
		Mean IRR^a^	2.5 PCT	97.5 PCT	Mean IRR^b^	2.5 PCT	97.5 PCT
Hyperactivity subscale	Phenols/parabens	0.92	0.79	1.04	1.12	0.97	1.29
	Phthalate metabolites	**1.11**	**1.00**	**1.23**	**1.14**	**1.06**	**1.26**
	Pesticide metabolites	1.03	0.95	1.11	0.95	0.89	1.03
	Trace elements	0.99	0.90	1.10	1.02	0.91	1.13
	Total mixture	1.04	0.87	1.18	**1.15**	**1.03**	**1.29**
Hyperactivity/impulsivity subdomain	Phenols/parabens	0.91	0.77	1.06	1.08	0.94	1.25
	Phthalate metabolites	**1.13**	**1.02**	**1.27**	**1.20**	**1.09**	**1.32**
	Pesticide metabolites	1.02	0.94	1.14	0.97	0.89	1.04
	Trace elements	1.01	0.89	1.15	1.05	0.93	1.22
	Total mixture	1.07	0.93	1.20	**1.21**	**1.05**	**1.43**
Inattention subdomain	Phenols/parabens	0.93	0.81	1.05	**1.13**	**1.00**	**1.28**
	Phthalate metabolites	1.05	0.98	1.12	**1.08**	**1.01**	**1.17**
	Pesticide metabolites	0.99	0.93	1.05	0.97	0.91	1.03
	Trace elements	0.98	0.88	1.08	0.99	0.90	1.09
	Total mixture	0.99	0.88	1.10	**1.11**	**1.01**	**1.25**

a WQS regression models were adjusted for child’s sex, birth year, and age at assessment, parity, parental education, maternal metabolic conditions, and diagnosis.

b WQS regression models were adjusted for child’s sex, birth year, and age at assessment, parity, parental education, and maternal metabolic conditions.

**Table 3:** Summary table of associations between each chemical or mixtures and ABC Hyperactivity subscale and two subdomain scores among all children and children with ASD.

Outcome	Chemical class	All (*n* = 574)		ASD (*n* = 232)	
		Each chemical^a^	Mixture^b^	Each chemical^a^	Mixture^b^
Hyperactivity subscale	Phenols/parabens	DHB34 (−) TCS (−)			
	Phthalate metabolites	MECPP (+) MEHHP (+)	MNBP (+) MHPP (+) MCINP (+)	**MECPP (+)** **MEHHP (+)** MBZP (+)	MNBP (+) MHPP (+) MBZP (+)
	Total mixture				DHB34 (+) MNBP (+) MHPP (+) MECPP (+) MEHHP (+)
Hyperactivity/impulsivity subdomain	Phthalate metabolites	**MECPP (+)** MEHHP (+) MNBP (+)	MNBP (+) MHPP (+) MCINP (+)	**MECPP (+)** **MEHHP (+)** **MBZP (+)** **MNBP (+)** **MCINP (+)**	MNBP (+) MHPP (+) MBZP (+)
	Total mixture				DHB34 (+) MNBP (+) MHPP (+) MECPP (+) MEHHP (+) MIBP(+) MBZP (+) Cd (+)
Inattention subdomain	Phenols/parabens	**TCS (−)**		OHMEP (+) PRPB (+)	DHB34 (+) ETPB (+) PRPB (+) BUPB (+) BPP (+) ВPAP (+)
	Phthalate metabolites			MIBP (+)	MNBP (+) MHPP (+) MIBP(+) MECPP (+)
	Total mixture				DHB34 (+) MNBP (+) MHPP (+) MECPP (+) MEHHP (+) MIBP(+) MBZP (+) Cd (+)

a Associations with significant associations are presented. Item in bold indicates significance even after FDR correction. (+) represents increased IRR and (−) represents decreased IRR.

b Possible contributors of mixtures that have significant associations with outcomes are presented. (+) represents increased IRR and (−) represents decreased IRR.

## Data Availability

Lab and epidemiological data are hosted at the Human Health Exposure Analysis Resources (HHEAR) Data Center Repository (https://hheardatacenter.mssm.edu/).
